# Biomechanical sensing of *in vivo* magnetic nanoparticle hyperthermia-treated melanoma using magnetomotive optical coherence elastography

**DOI:** 10.7150/thno.55333

**Published:** 2021-03-23

**Authors:** Pin-Chieh Huang, Eric J. Chaney, Edita Aksamitiene, Ronit Barkalifa, Darold R. Spillman, Bethany J. Bogan, Stephen A. Boppart

**Affiliations:** 1 Beckman Institute for Advanced Science and Technology, University of Illinois at Urbana-Champaign, USA.; 2 Department of Bioengineering, University of Illinois at Urbana-Champaign, USA.; 3 Department of Electrical and Computer Engineering, University of Illinois at Urbana-Champaign, USA.; 4 Carle Illinois College of Medicine, University of Illinois at Urbana-Champaign, USA.; 5 Cancer Center at Illinois, University of Illinois at Urbana-Champaign, USA.

**Keywords:** magnetic hyperthermia, iron oxide nanoparticles, cancer, elastography, optical coherence tomography

## Abstract

**Rationale:** Magnetic nanoparticle hyperthermia (MH) therapy is capable of thermally damaging tumor cells, yet a biomechanically-sensitive monitoring method for the applied thermal dosage has not been established. Biomechanical changes to tissue are known indicators for tumor diagnosis due to its association with the structural organization and composition of tissues at the cellular and molecular level. Here, by exploiting the theranostic functionality of magnetic nanoparticles (MNPs), we aim to explore the potential of using stiffness-based metrics that reveal the intrinsic biophysical changes of *in vivo* melanoma tumors after MH therapy.

**Methods:** A total of 14 melanoma-bearing mice were intratumorally injected with dextran-coated MNPs, enabling MH treatment upon the application of an alternating magnetic field (AMF) at 64.7 kHz. The presence of the MNP heating sources was detected by magnetomotive optical coherence tomography (MM-OCT). For the first time, the elasticity alterations of the hyperthermia-treated, MNP-laden, *in vivo* tumors were also measured with magnetomotive optical coherence elastography (MM-OCE), based on the mechanical resonant frequency detected. To investigate the correlation between stiffness changes and the intrinsic biological changes, histopathology was performed on the excised tumor after the *in vivo* measurements.

**Results:** Distinct shifts in mechanical resonant frequency were observed only in the MH-treated group, suggesting a heat-induced stiffness change in the melanoma tumor. Moreover, tumor cellularity, protein conformation, and temperature rise all play a role in tumor stiffness changes after MH treatment. With low cellularity, tumor softens after MH even with low temperature elevation. In contrast, with high cellularity, tumor softening occurs only with a low temperature rise, which is potentially due to protein unfolding, whereas tumor stiffening was seen with a higher temperature rise, likely due to protein denaturation.

**Conclusions:** This study exploits the theranostic functionality of MNPs and investigates the MH-induced stiffness change on *in vivo* melanoma-bearing mice with MM-OCT and MM-OCE for the first time. It was discovered that the elasticity alteration of the melanoma tumor after MH treatment depends on both thermal dosage and the morphological features of the tumor. In summary, changes in tissue-level elasticity can potentially be a physically and physiologically meaningful metric and integrative therapeutic marker for MH treatment, while MM-OCE can be a suitable dosimetry technique.

## Introduction

Magnetic nanoparticle hyperthermia (MH) is an emerging cancer thermotherapy with desirable merits such as remote triggering of heat induction, selective killing of tumors, and being minimally invasive. By manipulating the interaction between the magnetic nanoparticles (MNPs) within tumor and the external alternating magnetic field (AMF) operating at 50 kHz - 15 MHz 1-3, MH has raised extensive interest in the nanomedicine and radiation oncology communities. MNPs have been well known for their small, biocompatible, and biodegradable nature, and with further engineering, have enabled diagnostic and therapeutic applications. MNPs can be employed for tumor targeting, magnetic resonant imaging (MRI) contrast enhancement, drug delivery, and treatment of iron deficiency (approved by Food and Drug Administration in the United States) 4-6, and thus have an attractive potential for serving as a theranostic platform. MH therapy was achieved by exploiting the heat induction property of the MNPs either by the hysteresis or relaxation loss mechanism 6, and this technique has progressed greatly ever since its first demonstration in the 1950s 7.

Tumors are known to be more thermally sensitive than normal tissues due to their reduced ability in dissipate heat 8. With the application of thermal energy, both direct cytotoxic effect and indirect damage to the tumor cells can occur 1, 8, 9. MH therapy can demolish cellular integrity, inhibit DNA replication and repair enzymes, and activate an anti-tumor immune response. In addition, MH can also induce re-oxygenation, and reverse drug resistance, making it an important adjuvant treatment that enhances radio- and chemotherapy responses 1, 2, 9. Demonstrated in skin 10-13, prostate 14, intracranial 15, and mammary 16 tumors, MH therapy has induced tumor cell death, as well as therapeutic effects such as tumor regression and a prolonged survival rate. In the 2000s, clinical trials of MH alone or with external radiotherapy were successfully carried out on prostate cancer and glioblastoma patients in Germany 17, 18. Since then, efforts in clinical investigations of MH therapy have continued and expanded, with the United States recently beginning clinical trials in 2019 2.

One major challenge of MH is the lack of a physiologically meaningful thermal dosimetry method to appropriately measure and monitor dosage. MH therapy often claims to have the advantage of selectively killing only the tumor cells around the internal heating MNPs. However, in practice, it is difficult to ensure that surrounding healthy tissues remain unharmed. Traditionally, temperature-based metrics provide a convenient means of thermal dosage estimation. However, such metrics were formulated based on the cytotoxic effects observed mainly from *in vitro* studies or normal tissues, and may be subject to thermal sensitivity variations, multiple dose effects, and cannot directly reflect the tumor physiology under treatment 19-21. In addition, physiological changes could also be induced with nanoscale temperature rise as well 22, 23, which may not be detected from macroscopic temperature measurement. In addition, there is a lack of tools available for accessing the depth-resolved temperature profile within living tissues, which is disadvantageous for deep-seated tumors. While radiologic imaging technologies such as x-ray CT and MRI have been used to assess these temperature profile changes 24, 25, these are more indirect measures, as they rely on temperature changes altering the image properties of the tissue, which may not directly reflect physiological changes or be linearly proportional to the thermal dose. In summary, it is highly desirable to have an alternative or complementary metric that (1) is biophysical and physiologically informative and directly related to the MH-induced thermal dosage, and (2) can be measured with tools that probe tissue-level responses with high precision. It would be even more desirable if both aspects could be achieved via a theranostic platform.

Biomechanical properties, physical parameters associated with cellular and molecular level structural organization within tissues, can be regarded as integrative biomarkers for the tumor microenvironment and any associated pathophysiology 26, 27. Typically assessed for the diagnosis of cancer (e.g. the breast tumor “felt” stiff when being palpated 28; melanoma tumor appears stiffer than the surrounding skin 29), tissue elasticity can potentially be correlated with cancer therapeutic outcomes as well. During thermotherapy, cell membrane integrity can deteriorate, and protein folding, unfolding, or irreversible denaturation may occur 8, 9, all of which can affect tissue biomechanics 30-35. Observed on normal livers and muscles, tissue stiffness can change linearly with the thermal energy and exponentially with regard to conventional temperature-based dosage metrics 32, 35. Previously, ablative therapy was also shown to induce stiffening in prostate tumor and soft tissue sarcoma 33, 35. Recently, an interesting finding of softened breast and colon tumors was observed after chemotherapy, where dystrophic, edema, and necrotic cells all contributed to the decrease in elasticity, making tissue biomechanics a potential predictor of a complete pathologic response 36-39. Therefore, one important aim of this study is to examine the novel potential of stiffness-based therapeutic markers for *in vivo* MH therapy.

Elastography provides a means for tissue-scale, cross-sectional stiffness assessment via ultrasound imaging, MRI, or optical coherence tomography (OCT), among which OCT has the highest spatial resolution and displacement sensitivity 40-42. Magnetomotive optical coherence elastography (MM-OCE) achieves stiffness sensing by magnetically inducing nanometer tissue motions and detecting them via phase-sensitive OCT 43-45. With the deployment of MNPs into the tissues and the induction of a magnetic field gradient with a nearby coil, dynamic magnetomotive force is introduced, and thus modulates the tissue microenvironment immediately adjacent to the MNPs. The detection of the modulated “magnetomotion” enables the imaging of the distribution of MNPs, which is performed by magnetomotive OCT (MM-OCT) 46-49. As a functional extension, the viscoelasticity of the specimen can be extracted as MM-OCE further analyzes the temporal characteristics of the magnetomotion (e.g. mechanical resonant frequency 44, 50, 51, natural frequency 43, 52, or elastic wave propagation velocity 35, 45).

Both MM-OCT and MM-OCE techniques have demonstrated great potential for oncological applications. MM-OCT can track the location of directly injected MNPs in skin tumors 53 as well as visualize *in vivo* mammary tumor sites targeted by the use of antibody-conjugated MNPs 54, 55. MM-OCE, with the capability of differentiating tissue viscoelasticity, can visualize breast tumor margins by introducing mechanical contrast into OCT images 50. Mechanical resonance frequency is a biomechanical parameter that is proportional to the square root of Young's modulus, a longitudinal stiffness measure, for an elastic medium. It can be extracted from spectroscopic MM-OCE and indicates the collective elastic property of a tissue specimen with widely dispersed MNPs (as typically seen for MH therapy). Previously, spectroscopic MM-OCE has demonstrated its feasibility in detecting MH-induced stiffening of* ex vivo* chicken breast laden with passively diffused MNPs, which act both as heating sources in MH therapy as well as perturbative mechanical agents in MM-OCE 34. With the rising potential of MM-OCE for detecting the biomechanical response of tissues, our second aim is to investigate the feasibility and potential of using MM-OCE as a dosimetry tool for stiffness sensing of MH dosage delivered to tumors in living animals.

To provide a physically and physiologically meaningful biomarker to assist MH dosimetry in cancer treatment via a theranostic platform, a stiffness-based metric characterized by MM-OCE imaging was proposed in this study and demonstrated for the first time in an established *in vivo* melanoma tumor bearing mouse model. Melanoma is the fifth most common type of cancer in human among men and sixth most common cancer among women. When cutaneous melanoma is diagnosed at an early stage, patients who undergo surgical excision of the primary tumor can expect 5-year survival rates as high as 97%. However, at least 13% of melanoma patients already have metastases to regional or distant sites by the time they are diagnosed with the disease 56. Since metastatic melanoma is resistant to most traditional cancer therapies, alternative and novel curative strategies should be explored. Previously, a combination of hyperthermia and radiotherapy was shown to increase the complete response rate of malignant melanoma on human patients as compared to that treated with radiotherapy alone 57-59.

Here we demonstrate that the same MNPs, deployed to the tumor as MH therapeutic agents to produce heat, could also be detected with MM-OCT and be leveraged to also function as dosimetric agents that mechanically perturb the surrounding tumor microenvironment and enable stiffness assessment with MM-OCE. This study will help to not only fundamentally understand the biological effects caused by MH heating, but also the changes in biomechanical properties and their association with the temperature rise and the cellular and molecular (protein) composition of the tumor, validated by histological techniques.

## Materials and Methods

### Ethics statement

This study was conducted under a protocol reviewed and approved by the Institutional Animal Care and Use Committee (IACUC) at the University of Illinois at Urbana-Champaign (Illinois, USA).

### Tumor cell line and animal handling

A total of 14 wild-type mice (C57BL/6) aged 4.4 ± 1.2 months (mean ± STD) were inoculated with 2×10^6^ mouse melanoma B16F10 cells (ATCC, Virginia) by intradermal injection in the left flank, where the tumor grew over 14.2 ± 3.1 days (mean ± STD). Prior to the experiment, the hair near the tumor site was shaved and plucked. Isoflurane (1-3%) was administered to the mice through a nose cone during the entire experimental procedure, and a heating pad was placed underneath the mice during MM-OCT and MM-OCE imaging.

### Magnetic nanoparticles (MNPs) and delivery

Dextran-coated iron oxide nanoparticles were prepared according to 60 in order to improve their solubility in water. In brief, for every 10 mg of MNPs (maghemite Fe_2_O_3_, Sigma #544884), 10 mg dextran was added, and the mixture was resuspended in 20 ml of 0.5 M sodium hydroxide (NaOH) solution. Subsequently, the entire solution was sonicated for 1 - 2 h and dialyzed (Spectra/Por 4 Dialysis Trial Kit, Repligen) for > 24 h in distilled water so that excess dextran was removed. A total of 100 µl dextran-coated maghemite nanoparticle solution (~70 mg/ml in sterile phosphate buffered saline) was directly injected into the melanoma tumor using a 28-gauge hypodermic syringe and an injection rate of approximately 10-20 µl/min. Overall, 3-5 localized injection sites were chosen for each tumor. Magnetomotive imaging and MH treatment were performed after a diffusion time of at least 1 h after the intratumoral MNP delivery.

### Magnetic nanoparticle hyperthermia setup and temperature measurement

**Figure 1:**
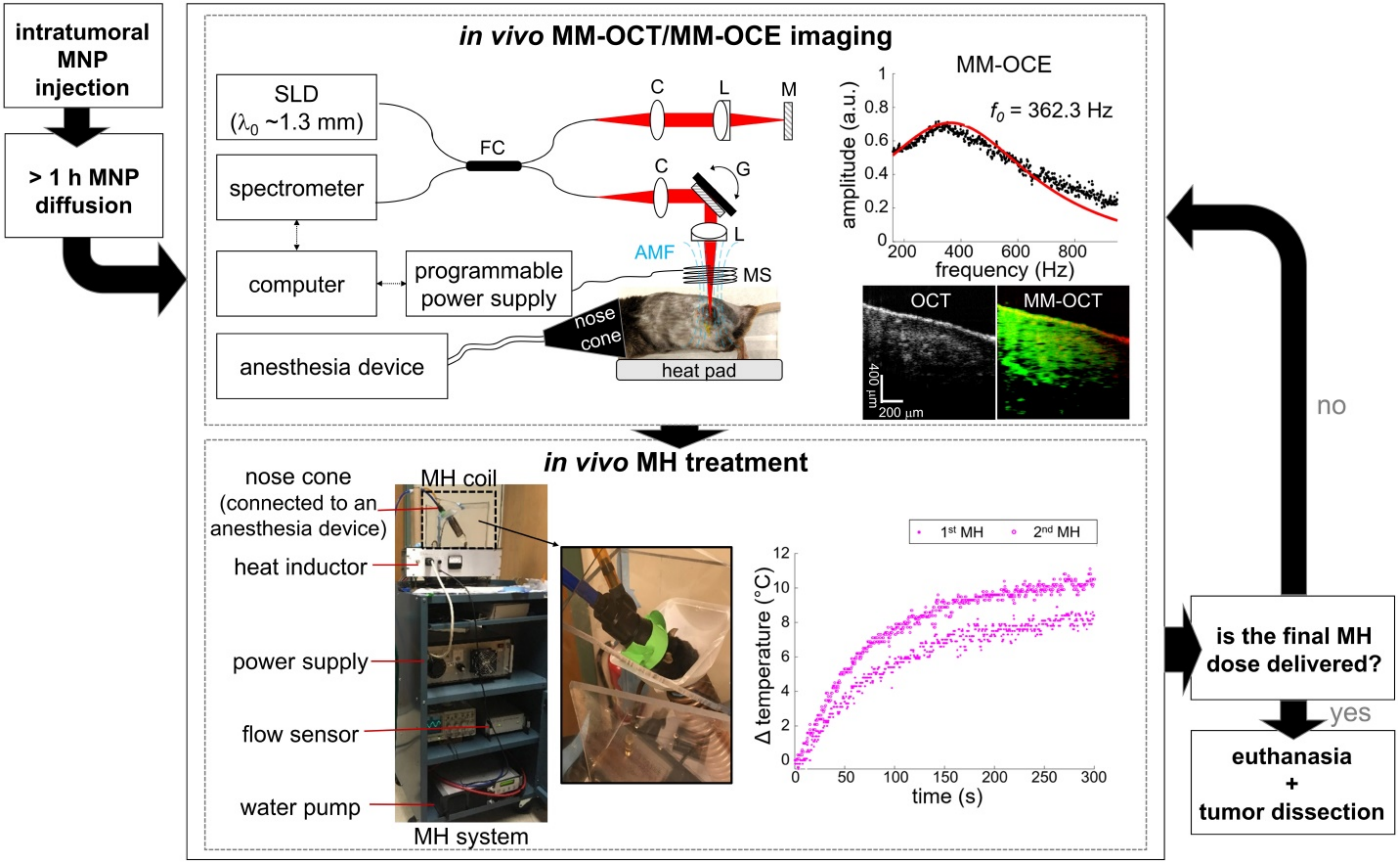
Experimental flowchart of MM-OCE-based stiffness sensing of *in vivo* MH-treated melanoma-bearing mice.

A customized, portable MH system 34 was utilized in this study (**Figure 1**). A copper-based hollow pipe coil, connected to a power supply and a heat inductor, generated an AMF oscillating at 64.7 kHz with a field strength of 61.2 kA/m (768.5 G) and 0 kA/m for the MH-treated and the control group, respectively. The MH coil has a total of 13 turns over 10 cm in length (1.3 turns per cm) and an inner diameter of 3 cm. Similar to other MH studies 11, 14, the control tumors were injected with MNPs but not AMF-treated. This also allows the biomechanical response of the non-treated tissues to be measured with MM-OCE 34. It is also worth noting that AMF exposure alone does not induce noticeable therapeutic effect on non-MNP-laden tissues 13, 16. To prevent overheating of the coil, chilled water was pumped and continuously circulated through the pipe coil and the water flow was monitored by a flow sensor. For the MH treatment, an animal was placed in a plastic holder located in the coil, and either a 5 min or a 9 min treatment was provided for 1 or 2 times. Additionally, a fiber-optic temperature sensor (OptoTemp 2000, MicroMaterials, Florida) was placed on the tumor mass to monitor its surface temperature during the MH treatment.

### Magnetomotive optical coherence tomography and elastography (MM-OCT & MM-OCE)

MM-OCT and MM-OCE measurements were conducted to validate the presence of MNPs in tumors and characterize the stiffness changes, respectively. MM-OCT/MM-OCE measurements were performed before and after each MH treatment (within 25 min after turning off the AMF).

A spectral domain OCT system with a superluminescent diode (wavelength 1325 ± 50 nm) light source provided an axial and transverse resolution of 8 µm and 16 µm, respectively, and was used with the magnetomotive setup for MM-OCT/MM-OCE measurements 35. The main additional component was an electromagnetic coil (inner and outer diameter were 6 mm and 20 mm, respectively), enclosed in a chilled-water-filled chamber, that induced a maximum magnetic field strength of 170 G. The coil was placed in the sample arm of the OCT system, right above the MNP-laden tissue, to induce magnetomotion. Subsequently, the modulated displacement was measured from the backscattered OCT light passing through the coil bore (diameter 2 mm). The modulation of the AMF was controlled by a programmable power supply, which connected directly to the coil. The dynamic tissue response was extracted from the phase term of the complex OCT data. The common mode displacement stability was measured to be 0.14 nm in air. MM-OCE measurements were performed on 4-6 locations near the same tissue site for each condition (before, after 1^st^, and after 2^nd^ MH treatment), while real-time OCT imaging was also performed to visually assist with image registration within a region of interest marked on the skin with surgical ink. At each location, 3-6 repetitive measurements were conducted.

Oversampled B-scan and M-mode images were acquired for MM-OCT and MM-OCE, respectively, as described previously 44, 48. For both signals, a total of 4096 A-lines were collected with a line scan rate of ~3 kHz. For MM-OCT, image acquisition was made over a transverse range of ~2 mm as a sinusoidal magnetomotive modulation was provided at a single frequency ≤ 0.7 kHz. For MM-OCE, interferometric signal was collected at the same spatial location over time as a chirped AMF excitation (10 Hz to ~1 kHz) was applied. Data processing of MM-OCT/MM-OCE also followed the standard approach 34. In brief, tissue motions were obtained from the unwrapped phase difference between consecutive A-lines or temporal measurements of complex OCT data. For MM-OCT, bandpass filtering was performed at the modulation frequency, and the magnitudes of the MM displacements at each spatial location were obtained from the envelope of the complex analytical signals. For MM-OCE, the phase signal was extracted from the complex signal averaged over 3 rows around the depth with the maximum signal-to-noise ratio (SNR). Subsequently, a mechanical spectrum was computed by Fourier transform, which shows the displacement amplitude as a function of frequencies. A Lorentzian fit was performed on the mechanical spectrum to extract the resonant frequency (*f_0_*), where the maximum displacement occurs. The main sources of background noise, respiration and cardiac motion, if any, were suppressed by neglecting the lower frequency regime during fitting. Only the data with an R-squared value (R^2^) > 0.5 was included for statistical analysis.

The resonant frequency (*f_0_*) extracted from the mechanical response is proportional to the square root of the Young's modulus (*E*), a stiffness measure, (i.e. *f_0_* ∝ 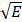) assuming negligible variations in mass and geometry for an elastic medium 34, 44, 61. Therefore, the alteration of tissue stiffness after MH treatment can be indicated by the percentage change in the squared resonant frequency (*f_0_^2^*) calculated with respect to the pre-treatment data, which is the main metric utilized throughout this study. For statistical analysis, a paired Student's t-test was performed among the *f_0_* values with a significance level of 0.01.

### Histological examination and cellularity quantification

Histological examination was performed on the melanoma tumors from all 14 mice, where 11 mice have undergone AMF treatments and 3 mice were non-treated (control). After the completion of all MH treatments (1 or 2 doses), the mice were immediately euthanized with carbon dioxide (CO_2_) gas. Subsequently, the MNP-laden melanoma tumor, as well as the spleen, were dissected and immersed in formalin for at least 8 h for fixation. The spleen is part of the reticuloendothelial system (RES) where the macrophages can uptake the MNPs during the clearance process 62. To ensure that the AMF applied would damage only the MNP-injected tumor sites, the viability at other organs that may potentially be at risk (e.g. spleen, where MNPs accumulated naturally) was also evaluated. The specimens went through standard tissue preprocessing steps prior to being paraffinized and sectioned into 5 µm thick slices with a microtome (Leica RM2255, Leica Microsystems GmbH, Wetzlar, Germany). Afterward, histological staining was performed, followed by qualitative/quantitative slide micrograph analysis.

To verify the presence of intratumorally-injected MNPs, iron-oxide staining (HT20, Sigmal-Aldrich) was also performed. To visualize and differentiate collagen fibers in tissue sections, the slides were stained with Masson's trichrome (MT) stain or picosirius red (PR) stain kits following the recommended manufacturer's protocols (Polysciences, Inc., Warrington, PA). The stained histological sections were imaged and digitized under brightfield microscopy (Nanozoomer, Hammamatsu). Moreover, PR-stained slides were also imaged under polarized light so that fibrillar collagen proteins (type I and type III collagen), with their birefringence property, could be visualized with better specificity 63. Additionally, cell death was assessed by TUNEL assay (*In situ* Cell Death Detection Kit, Fluorescein, Sigmal-Aldrich). The TUNEL-stained slides were coverslipped with a mounting medium containing DAPI nuclear counterstain (H-1500, Vector Laboratories). The TUNEL label and DAPI counterstain were imaged with an excitation/emission at 470/525 nm and 365/445 nm, respectively, with built-in filter sets in an inverted phase contrast fluorescent microscope (Zeiss Axio Observer D1, Carl Zeiss Microscopy, White Plains, NY).

To evaluate the tissue sample cellularity, color-based quantification was made based on the digitized PR-stained images. An overall cellularity metric was defined as (1 - overall cytoplasm area/overall tissue area). In addition, a local cellularity metrics was defined as (1- local cytoplasm area/local tissue area). The overall area was computed from the entire image of the tumor sections. The local area was calculated near the center of mass of MNP distribution with a radius equals to the maximum value of the interquartile ranges of the MNP distributions in the vertical and horizontal directions. Cytoplasm area was quantified as the yellow area in PR stained sections 64, which was extracted from the “b” channel in the “l, a, b” color space. Tissue area was computed via binary thresholding, which excluded the dark-brown areas that represented MNPs and the white background. The red tissue region indicates the presence of both fibrillar (type I and type III) and nonfibrillar (type IV) collagens, as well as keratohyalin granules of cornified epithelia, and mucous glands 63, 65. Interestingly, the PR-stained, color-based cellularity metrics correlated well with the nuclei density detected, where the PR-stained yellow region often corresponded to the region where nuclei was less densely packed (**Supplementary Figure S1**). Therefore, the yellowness in PR-stained histology may indicate areas where cytoplasm has been released from the intracellular space and into the interstitial space, potentially due to cell lysis or necrosis following MH treatments.

## Results and Discussion

**Figure 2:**
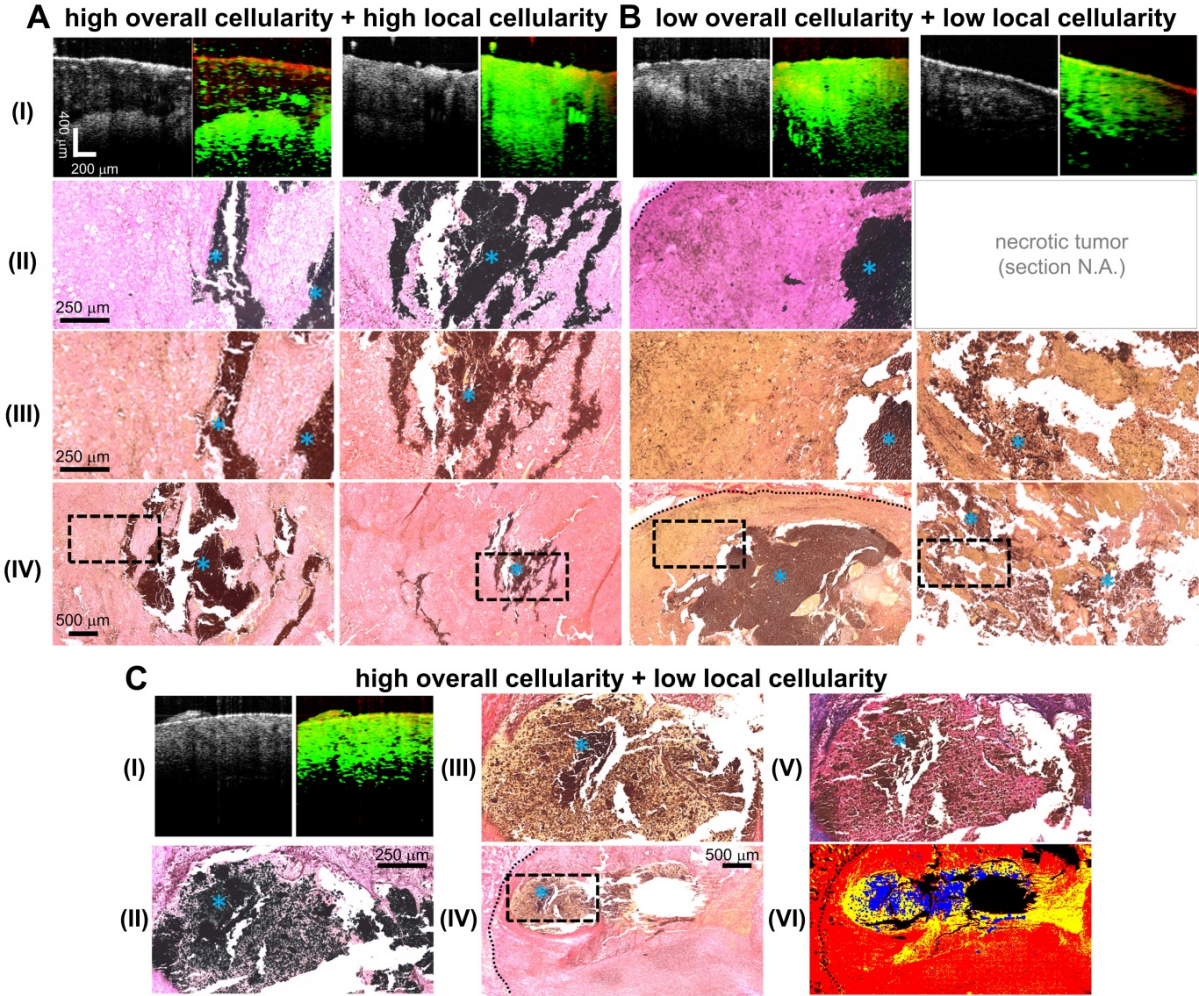
** Representative images of MNP-laden murine melanoma tumors with differential degrees of overall and local cellularity. A-C. I.** Simultaneously-acquired *in vivo* OCT (*left*) and MM-OCT (*right*) depth-resolved cross-sectional images of melanoma tumor tissue with high overall and local cellularity (A), low overall and local cellularity (B), or high overall and low local cellularity (C). MM signals are color-coded in green, which are overlaid on top of structural OCT images color-coded in red. **II.** The presence of intratumor MNPs (*blue asterisks*) confirmed with iron-oxide staining. **III.** High magnification photomicrographs of histological tumor sections stained with Picrosirius Red (PR). These enlarged images correspond to the areas indicated by the dashed black line boxes in the low magnification images **(IV). C.** The structures in a representative MT-stained slide section **(V)** strongly correlate with those detected by PR (III); the cytoplasm appears as (III) yellow or (V) pink. **VI** shows a representative masked image utilized for quantification of tissue cellularity, where the cytoplasm, non-cytoplasm, and MNP areas were coded in yellow, red, and blue, respectively. Histological images were all obtained under bright-field microscopy. Blue asterisks indicate MNPs. The dotted black lines delineate the tumor margin. One tumor was severely necrotic and a few of its histological sections were lost **(B, II)** as the tissue sections did not retain any structural integrity. As a result, they were shattered upon sectioning, or dissolved instantly when floated on the warm water bath, or weakly attached to the microscope slides, and hence were washed off easily.

Due to its aggressive growth and a common tumor model explored in MH studies 10, 11, 13, the B16-derived cell line was used in this melanoma MH research study. Successful tumor development was observed in all 14 mice. The presence of injected MNPs in the center of the tumor was observed in 13 out of 14 samples from the MM-OCT images, indicating successful MNP administration (92.86% accuracy). MNP introduction into the tissue was also validated by the Prussian blue iron oxide staining histology as shown in **Figure 2**. Observed from the iron oxide staining histology, one tumor-bearing mouse had little to no MNPs present in the tumor core (likely due to insufficient injection depth) and was thereby excluded from further analysis.

**Figure 3:**
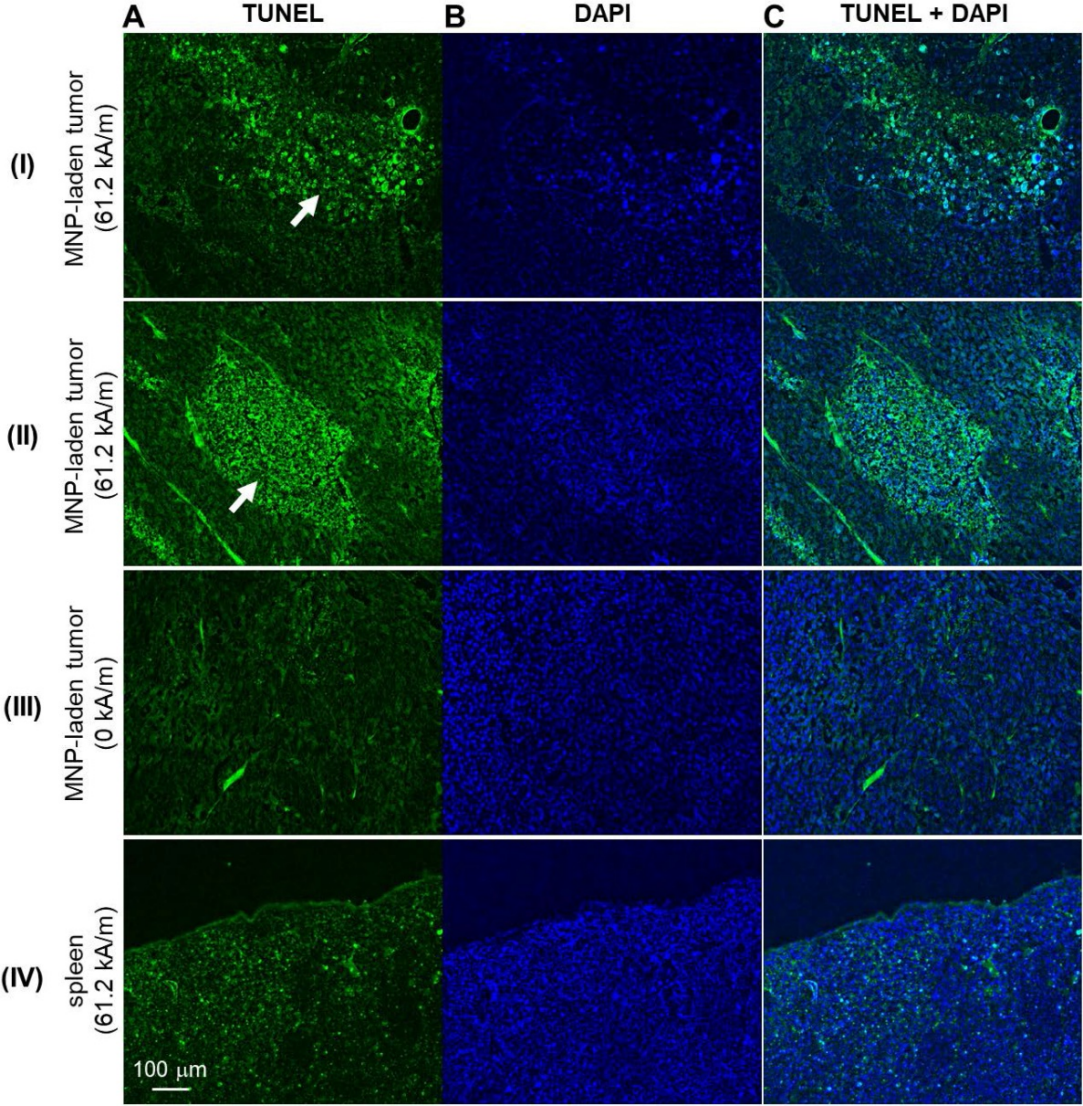
** The impact of an alternating magnetic field (AMF) on murine melanoma tumor and control organ (spleen) tissue/cell death.** Representative TUNEL-stained **(A)**, DAPI-stained **(B)**, and the overlaid/merged **(C)** images of histological tumor sections. MNP-injected tumors with **(I-II)** and without **(III)** AMF application. **IV.** Micrographs of TUNEL- and DAPI-stained spleen of the melanoma-bearing mice that underwent AMF application. White arrows indicate areas of cell death.

With the MNPs well-deposited to the tumor site, MH treatment was induced by applying 61.2 kA/m AMF strength to the experimental animals or 0 kA/m to the control animals. Subsequently, the MH-induced stiffness changes were probed by MM-OCE measurements. Significant temperature rise was only observed in the MH-treated group, with a maximum temperature change (ΔT_Max_) of 8.67 ± 3.50 ˚C. In contrast, the control group exhibited a slight decrease in temperature (ΔT_Max_ = -2.14 ± 0.63 ˚C), which was likely due to the chilled water circulating through the coil, as similarly observed in 10. The MH therapy successfully led to cell and tissue death, as evident by the fact that TUNEL signals were only observed from the MH-treated tumors (**Figure 3**). In contrast, a low level of TUNEL signals were detected from the tumors of the control mice (MNP injected to the tumors, AMF not applied). Additionally, a similarly low level of TUNEL signals were exhibited in the spleens of the AMF-treated mice (MNP injected to the tumors, AMF applied). Collectively, this indicates an MH-enabled selective killing at the MNP-injected tumor sites, as neither the non-treated tumors nor the AMF-treated healthy organs (where MNPs can naturally accumulate) manifested evidence of cell death.

**Figure 4:**
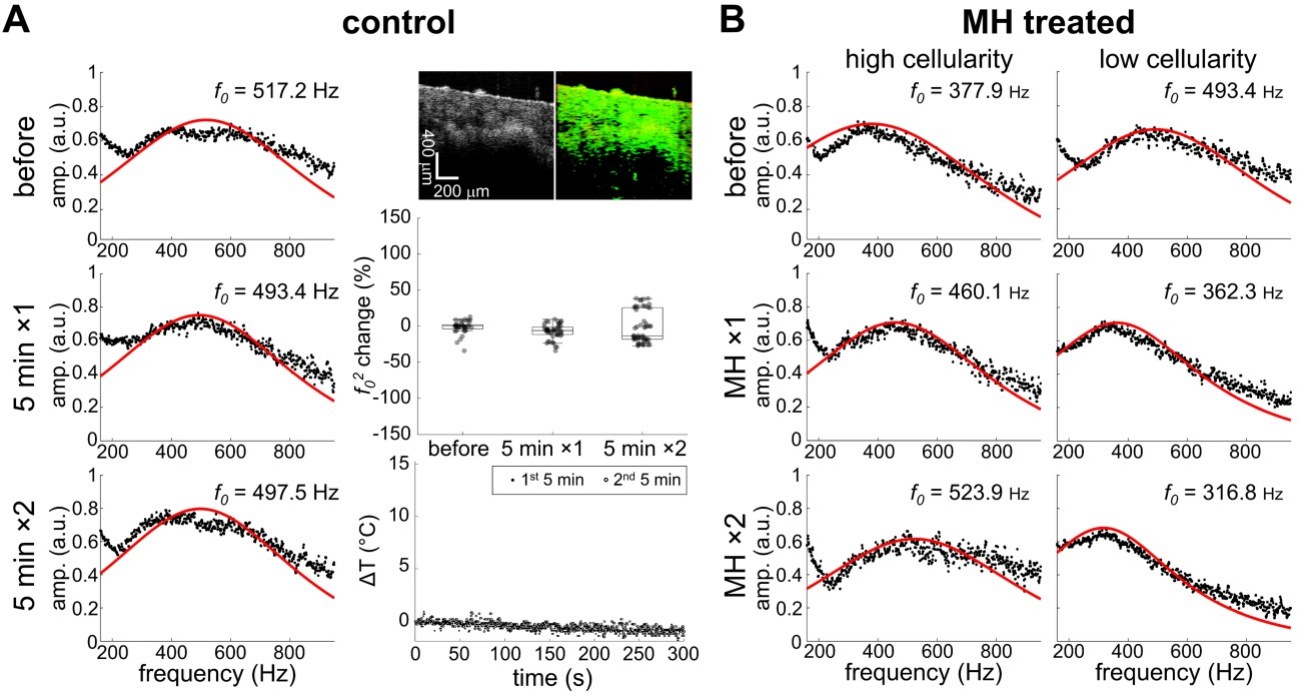
** Heat-induced stiffness changes following MH**. **A.** Control data (0 kA/m) shows negligible changes in temperature (ΔT), mechanical spectrum, and *f_0_^2^* percentage, where *f_0_* differences over time are minimal (N = 3). MM-OCT confirms the presence of MNPs. **B.** MH-treatment (61.2 kA/m) induced changes in the mechanical spectra.

Noticeable changes in the square of the resonant frequency (*f_0_^2^*) were only observed in the MH-treated group while the resonant frequency changes of the control group were negligible (**Figure 4**). This suggests that the stiffness changes originated from the induction of heat caused by the applied AMF. It is understood that the biomechanical changes of the tissues depend not only on the tissue composition, but also on the structural organization, and the resultant physical alterations at the cellular and molecular level. The overall elasticity can be affected by the thermally-altered conformation of the cells and proteins, both of which are involved in the cytotoxicity mechanisms in hyperthermia. In fact, the cytoskeletons are heat-sensitive and the thermal energy for cell death induction is in the similar range of that for cellular protein denaturation 1, 9. Here, we investigated the MH-induced stiffness change of melanoma tumors by considering the effects of tissue and tumor composition, including cellularity and collagen protein content.

### Tissue softening observed on tumors with lower local cellularity

First, the association between tissue stiffness and cellularity was investigated. The median overall and local cellularity of the melanoma tumor specimens were quantified to be 83.6% and 85.5%, respectively, based on their histological appearance. The tumor specimens were categorized into two cellularity groups (high or low) based on the median local cellularity measurements, as regions in immediate proximity to MNPs are in the lethal region after MH therapy. Note that 2 out of the 11 treated mice were excluded from the quantitative cellularity analysis as one showed absence of MNPs at the tumor core (as mentioned earlier) and the other had MNPs trapped in a highly heterogeneous environment (**Supplementary Figure S2**). The main investigation was carried out on the animals that underwent 5 min MH treatments (N = 7, where 4 and 3 tumors had low and high cellularity, respectively). Further exploration on the influence of a prolonged 9 min MH treatment was performed as well (N = 2, with 1 high and 1 low cellularity).

**Figure 5:**
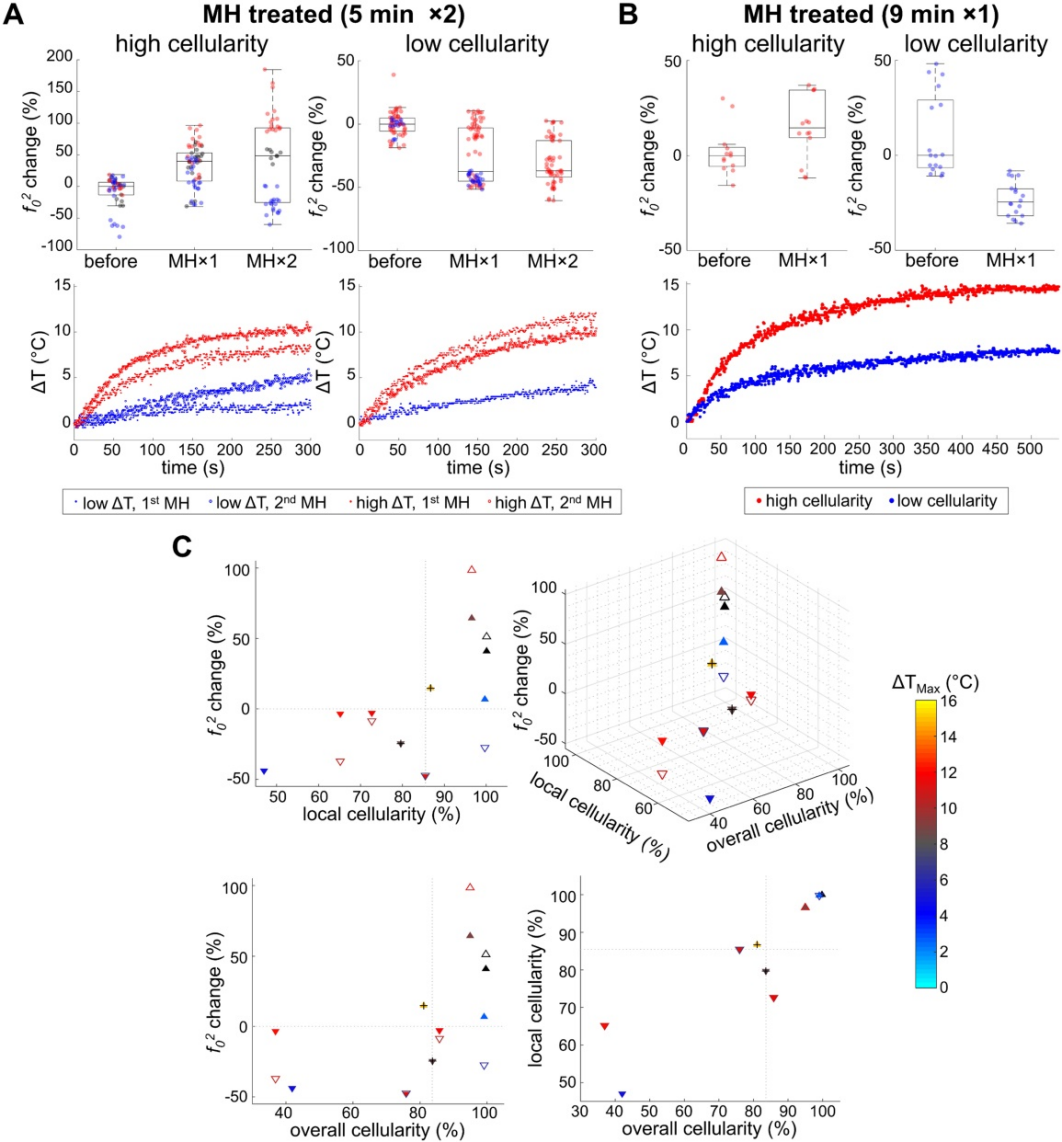
** Heat-induced stiffness changes and their association with tumor cellularity and temperature rise**. **A.** With 5 min MH treatment, consistent softening (decreased *f_0_^2^*) was observed from the low cellularity samples, despite whether a high ΔT (N = 3) or low ΔT (N = 1) is achieved. For highly cellular samples, a reversible *f_0_^2^* alteration was seen for low ΔT (N = 1), while consistent stiffening (increased *f_0_^2^*) was observed otherwise (N = 2). **B.** With 9-min MH treatment, similar results were observed from samples with both high and low cellularity (N = 1 for each group). **C.** Scatter plots of the MH-treated results (5 min MH data are marked with triangles and 9 min MH data are additionally marked with a cross symbol). Triangles pointing up/down indicate stiffening/softening. Solid or hollow triangles indicate the 1^st^/2^nd^ treatments. Maximum temperature rise (ΔT_Max_) is color-coded for each scatterer, while black indicates unmeasured temperature. Gray dashed lines indicate the median values of either overall or local cellularity.

Our data presented in **Figure 4B** and **Figure 5** indicate that MH resulted in decreased *f_0_^2^* (*f_0_^2^* change < 0), or decreased elasticity, for the tissues exhibiting lower local cellularity (< 85.5%). A statistically significant difference was found between the *f_0_* values obtained before, after the first dose, and after the second dose of 5 min MH treatments, where a corresponding median *f_0_^2^* change of -37.4% (N = 4) and -36.8% (N = 3) were observed (**Supplementary Table S1**). The low local cellularity was likely a result of necrosis, where the breakdown of the cell membrane integrity can lead to the release of cytoplasm, making the PR-stained or MT-stained tissues appear more yellow or more pink, respectively (**Figure 2C**). Moreover, a low local cellularity also indicates the presence of cytoplasm near the internal heating MNPs, which potentially suggests heat-induced cell lysis. As morphologically observed in the literature 66, increased cell membrane fluidity can occur, and result in rupture of the plasma membrane upon heat deposition. In addition, necrosis-associated softening was detected in various tumors 36-38, and a decreased tumor cellularity can be utilized to indicate a good pathological response after chemotherapy 36. From our data, a decreasing *f_0_^2^* was consistently observed with consecutive applications of thermal doses. Both 5 min (N = 4) and 9 min (N = 1) MH treatments induced tumor softening, where a significant decrease of *f_0_^2^* (-24.6%) was also observed for the latter treatment.

In one particular tumor that underwent 5 min treatments, evidence of severe necrosis was observed when preparing the histological tissue sections (**Figure 2B, II**), which may imply chronic tumor necrosis even prior to MH treatment. The tissue sections of the tumor did not retain any structural integrity, and hence some sections shattered during sectioning, or dissolved immediately when floated on the warm water bath, or weakly adhered to the microscope slides, and hence were washed off easily. In this case, the tumor cellularity was extremely low (both the local and overall cellularity < 50%) and great softening (-43.7%) was induced with a small temperature rise (ΔT_Max_ = 4.9 °C) (**Figure 5C**). This potentially suggests that acute cell lysis could be induced more easily (without demanding much thermal energy) when chronic tumor necrosis (caused by deprivation of vascularization and nutrients 67) is present.

### Temperature-dependent stiffness changes observed in tumors with collagen fibers

**Figure 6:**
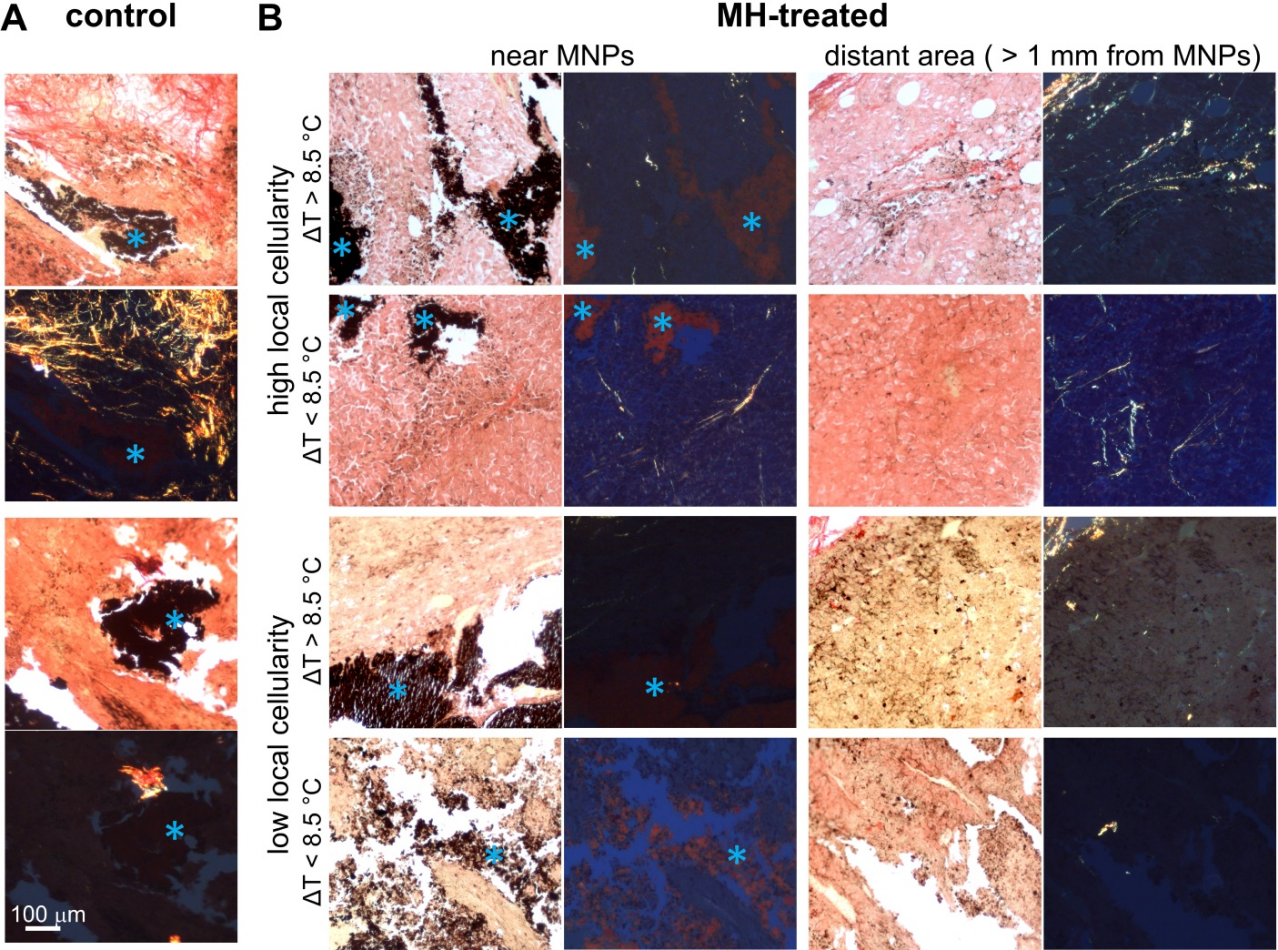
** Representative picrosirius red-stained melanoma tissue micrographs imaged under bright-field and polarized light. A.** The presence of collagen fibers near MNPs in control untreated mice melanoma tumor tissue. **B.** The absence of collagen fibers in MH-treated mice melanoma tumor tissue samples with low local cellularity. In contrast, collagen fibers are present in tumor tissue samples with high local cellularity. With ΔT_Max_ < 8.5 °C, collagen fibers are found both close to MNPs and distant from MNPs (> 1 mm from MNPs); with ΔT_Max_ > 8.5 °C, collagen fibers are found only at area farther away from MNPs. Blue asterisks in the bright-field microscopy images indicate MNPs. Note that 8.5 °C was selected as the reference value as it represents the mean ΔT_Max_ achieved in the highly cellular tumors after the 2^nd^ MH treatment (**Table S1**).

Next, we investigated the contribution of protein conformation to the biomechanical response of the MH-treated tumors. The histological examination of the melanoma tumor tissue images captured under polarized light showed randomly scattered collagen fibers (**Figure 6**). Overall, the presence of collagen fibers in each tumor mass was minimal, which is in agreement with previous statements indicating that melanoma tumors contain relatively little collagen 26, or may indicate that B16F10-cell derived tumors in particular may have a less differentiated phenotype 68. In addition, the amount of collagen fibers present was particularly low (0.28 ± 0.25% area is birefringent) in tumors with low cellularity (**Figure 6**).

From the highly cellular tumors (local cellularity > 85.5%), increased elasticity (indicated by the percentage changes of *f_0_^2^*) was observed in all samples (N = 3) after receiving the 1^st^ dose of 5 min MH treatment. Interestingly, while most of the highly cellular tumors also showed an increased elasticity after the 2^nd^ MH dose, one tumor had softened. Despite the relatively small sample size, the divergent elasticity changes (of tumors with similar level of cellularity) induced with the same MH treatments have suggested influences from additional factors. Exploration on the dependency on temperature rise and the collagen conformation form was subsequently made. In the scenario where a higher temperature rise (ΔT_Max_ ≥ 8.9 °C) was induced, the tumor showed consistent stiffening (64.2% after the 1^st^ dose and 98.7% after the 2^nd^ dose), and the corresponding polarized image detected the birefringence signals over a noticeable area only in the peripheral regions (4.1%) that were farther away from the MNP heating agents but not nearby (0.5%). Interestingly, one tumor sample showed an acute stiffening (+6.9%) followed by softening (-27.5%) when a small temperature elevation (ΔT_Max_ ≤ 5.9 °C) was induced, and the percentage area containing birefringence signals at an region distant from the tumor (2.5%) and that near the MNPs (1.4%) was at a similar level (**Figure 6**). Analyzed from all the highly cellular tumors, statistically significant changes were observed before treatment and after the 1^st^ dose, as well as before treatment and after the 2^nd^ dose. However, due to the deviating stiffness changes after the second MH dose (stiffening/softening was induced with a higher/lower temperature rise level, respectively), no statistical significance was observed between the tumors that were treated once versus twice across N = 3 samples (**Supplementary Table S1**).

Proteins are commonly known to have temperature-dependent structural and conformation forms, and hence will directly affect the mechanical properties of the tissue. At a lower temperature rise, protein unfolding/refolding can occur, and hence lead to tissue softening or a reversible mechanical effect. On the other hand, when the temperature is elevated significantly, irreversible protein denaturation can take place, and consistent stiffening will occur 30, 31. This hypothesis is further supported by the diminished birefringence signal near the MNPs, which is likely the result of protein/collagen denaturation near the heating sources (**Figure 6**) 69. For the 9 min MH application (N = 1), an increased *f_0_^2^* value (14.6%) was observed between the before versus after treatment scenarios from the same sample without statistical significance (*p* = 0.07). Although the 9 min treated tumor had a high local cellularity, the overall cellularity was slightly lower (81.1%), which likely explains the insignificant difference observed. Note that in general, birefringence signals (most commonly arising from ordered collagen fibers) were rare at the cytoplasm-rich regions, as shown in the samples with low cellularity (**Figure 6B**). Additionally, an interesting finding was obtained in a tumor where MNPs were present in a highly heterogeneous microenvironment, where location-dependent MM-OCE results was observed and histology showed the presence of MNPs at regions with both high and low cellularity, and both near or away from collagen structures (**Supplementary Figure S2**).

Overall, the elasticity of MH-treated melanoma was shown to be strongly influenced by the cellularity, collagen content and conformation, and the magnitude of the temperature rise (**Table 1**). Tumors with lower cellularity (and less collagen fibers) were softened by the MH therapy regardless of the amount of temperature rise, which is likely due to the increased amount of necrosis after thermal perturbation. For tissues with higher cellularity and more collagen fibers, the change in stiffness was further affected by the level of temperature elevation. With a lower temperature rise (ΔT_Max_ = 5.9 °C), tumor softening was observed, likely due to the unfolding process of the collagen fibers. With a greater temperature rise (ΔT_Max_ = 11.1 °C), tumors exhibited progressive stiffening, which is potentially the result of collagen cross-linking or aggregation of unfolded protein during an irreversible denaturation process 30, 31. It is also worth noting that even with similar induced temperature changes (**Figure 5A**); biological variations (tumor necrosis or protein conformational changes) can still result in different biomechanical changes. This suggests that temperature-based metrics alone do not reflect the biophysical and physiological changes in tumor. In contrast, the elastic biomechanical changes can serve as an integrated biomarker of the tumor microenvironment and represent the collective biophysical changes induced by MH doses. Although most of the tumors were treated with 5 min MH treatments (doses), the conclusions drawn can also be applied to the tumors that underwent a prolonged 9 min dose, suggesting similar effects of MH treatment with minute-scale durations.

## Conclusions

For the first time, we demonstrated dosimetry metrics reflective of biomechanical changes of the *in vivo* murine melanoma tumor as a result of MH cancer treatment. As a versatile platform with theranostic functionality, dextran-coated MNPs were injected into the melanoma tumor mass, detected and imaged by MM-OCT, driven to thermally perturb the tumor microenvironment in MH treatment, and exploited in MM-OCE as subtle vibration sources to assess the elasticity alterations of the MH-treated tumors. It was empirically discovered that the altered stiffness, probed by MM-OCE, can potentially be a therapeutic biomarker which carries integrative information about the heat-induced physical and physiological changes in tumors. In addition, the influence of tumor cellularity, as well as collagen denaturation, on the MH-induced stiffness changes were investigated. Empirical findings obtained from this investigation are beneficial for future applications related to stiffness-based MH dosimetry.

In this explorative study, softening of tumors were observed from all samples showing low cellularity (and less collagen fibers), which likely indicated heat-induced necrosis, where cytoplasm was released into the extracellular space as visualized by histology. In the highly cellular (also collagen-containing) tumors, while reversible stiffening occurred in one tumor following a mild temperature rise, most tumors have been irreversibly stiffened after the MH treatments as the temperature elevated sufficiently high and the birefringence signals diminished. This is potentially a result of collagen cross-linking or aggregation of unfolded proteins, as the tissue manifested no birefringence around the MNP heating sites; yet birefringence signals were seen in distant regions within other tumor regions, far away from the MNPs. The sample size of the investigation can be improved in the future. Nevertheless, the dependency of both the cellularity and collagen conformation forms on the heat-induced stiffness changes of *in vivo* melanoma tumors has allowed novel research opportunities from three perspectives. First, the biomechanics-based metric may be utilized to infer biological conditions, such as cellularity and protein denaturation or cross-linking, in melanoma tumors once a systematic investigation on a larger scale is conducted. Second, the stiffness change after MH therapy can potentially serve as an early treatment indicator. With the readily accessible imaging capability of polarization-sensitive OCT, the presence of collagen content in tissue or tumors may be assessed, with either hand-held probes or with a needle-based probe 70, 71. The evaluation or mapping of stiffness changes can conceivably be sensed with the incorporation of OCE into a handheld imaging probe 72, 73. Finally, the evidence of a dosage dependent outcome has indicated the potential feasibility of MM-OCE-based MH dosimetry in the future.

Theranostic functionality enabled by a combination of MNP-based treatments and the MM imaging modalities has been of research interest in recent years, as demonstrated for hearing augmentation 74 and MH dosimetry 34, 75. This work presents the first *in vivo* oncology application of a MM-imaging enabled theranostic platform. Previously, MH-treated chicken breast and porcine liver specimens exhibited a noticeable stiffening after MH, which is in contrary to our major findings from the melanoma tumor. Not only does this highlight the value of a tumor study, particularly one done *in vivo*, but it also emphasizes the importance of the fundamental understanding of the association between the pathophysiology and the heat-induced elastic changes. In fact, tumor softening after therapeutic intervention has been demonstrated in breast and colon tumors, which often indicates a complete pathological response 36, 38. In addition, thermal perturbation can also lead to acute or transient stiffening (within the same day of treatment) followed by softening (at days after treatment) in both epidermoid carcinoma and cholangiocarcinoma tissues, where depleted cancer-associated fibroblasts (often related to poor prognosis) induced by photothermal therapy contribute to tumor softening and regression 76, 77. Similar phenomena were observed here, albeit melanoma tumors are known to have minimal fibrillary protein networks 26, suggesting MM-OCE also has the potential to predict treatment outcome after therapeutic interventions.

Recently, an adjuvant cancer therapy was proposed which aims to thermally break the physical barriers (e.g. collagen stroma) of the tumor microenvironment (TME) and therefore facilitate drug penetration into the tumor 76-78. Hence, in addition to indicating tumor cell physiological changes (e.g. necrosis), stiffness-based metrics may also be utilized to monitor the modulating of the stroma around a desmoplastic solid tumor in this novel treatment. Previously, heat-induced remodeling of the tumor stroma has been demonstrated, where evidence of collagen denaturation/destruction was shown and the tumor softening was successfully characterized with ultrasound elastography 76, 77. Conceivably, the bulk tissue softening can also be sensed by MM-OCE, which can be potentially indicative of the heat-induced molecular-level changes including not only necrosis (directly related to tumor damage) but also denaturated/destructed stroma at the TME (facilitating adjuvant chemotherapy treatments). Interestingly, the alteration of the stroma rigidity itself may also modify the phenotypes of the tumor cells favorably 79, where the slackened tumor tissues have shown to be accompanied by tumor regression 76, 77.

In summary, this work has examined the potential of using a stiffness-based metric and biomarker to evaluate the heat induced therapeutic effect on tumor, and demonstrated the capability of using MM-OCE to successfully extract the biophysical features. Heat-induced softening was observed on tumors with lower cellularity (lower cell density and more cytoplasm released to the interstitial space) and a lack of collagen deposition, indicating potential necrosis. In contrast, tumor stiffening was observed after the first MH dose on tumors with higher cellularity and containing collagens, where a higher temperature elevation can induce protein crosslinking or irreversible denaturation. Collectively, MM-OCE-based assessment of MH treatment outcome offers new opportunities in theranostic applications for oncology, and can potentially enhance real-time, personalized therapies in the future.

## Supplementary Material

Supplementary figures and tables.

## Figures and Tables

**Table 1:** Summary of heat-induced elasticity change on *in vivo* melanoma tumors

	Δ*T_Max_*^†^	Presence of birefringence^‡^		*f_0_^2^* change	
		Distant from MNPs	Near MNPs	After 1^st^ MH	After 2^nd^ MH
Low cellularity	High	No	No	Decrease (↓)	Decrease (↓)
	Low	No	No	Decrease (↓)	Decrease (↓)
High cellularity	High	Yes^§^	No^§^	Increase (↑)	Increase (↑)
	Low	Yes	Yes	Increase (↑)	Decrease (↓)

†High and low Δ*T_Max_* indicate Δ*T_Max_* > 8.5 °C and Δ*T_Max_* < 8.5 °C, respectively. ‡Distant from and near MNPs indicate > 1 mm and < 1 mm from MNPs, respectively. §Diminished birefringence observed near the heating agents (MNPs) potentially suggests collagen crosslinking or aggregation of unfolded proteins during an irreversible denaturation process.

## Abbreviations

MH: magnetic nanoparticle hyperthermia
MNPs: magnetic nanoparticles
MM-OCT: magnetomotive optical coherence tomography
MM-OCE: magnetomotive optical coherence elastography
AMF: alternating magnetic field
MRI: magnetic resonant imaging
OCT: optical coherence tomography
STD: standard deviation
SNR: signal-to-noise ratio
MT: Masson's trichrome stain
PR: picosirius red stain
TUNEL: Terminal deoxynucleotidyl transferase dUTP nick end labeling
DAPI: 4',6-diamidino-2-phenylindole
