# Supplementary material for: Biomechanical sensing of in vivo magnetic nanoparticle hyperthermia-treated melanoma using magnetomotive optical coherence elastography
Source: Theranostics. 2021 Mar 23;11(12):5620–33. doi: 10.7150/thno.55333 (PMC8058715; doi:10.7150/thno.55333)
Supplement: Supplementary file 1 — Supplementary figures and tables. [file thnov11p5620s1.pdf]

Supplementary Material

Biomechanical sensing of *in vivo* magnetic nanoparticle hyperthermia treated melanoma tumor using magnetomotive optical coherence elastography

Pin-Chieh Huang[1,2], Eric J. Chaney[1], Edita Aksamitiene[1], Ronit Barkalifa[1], Darold R. Spillman, Jr.[1], Bethany J. Bogan[1], and Stephen A. Boppart[1,2,3,4,5]

[1]Beckman Institute for Advanced Science and Technology, University of Illinois at Urbana-Champaign, USA
[2]Department of Bioengineering, University of Illinois at Urbana-Champaign, USA
[3]Department of Electrical and Computer Engineering, University of Illinois at Urbana-Champaign, USA
[4]Carle Illinois College of Medicine, University of Illinois at Urbana-Champaign, USA
[5]Cancer Center at Illinois, University of Illinois at Urbana-Champaign, USA

Table S1. Stiffness changes after magnetic nanoparticle hyperthermia (MH) treatments

**Case**	**Local cellularity (%)**	**N[†]**	**ΔT_{Max} (°C)**		**f_0^2 change (%)**		**Statistical significance**		
			**After D1**	**After D2**	**After D1**	**After D2**	**B/D1**	**B/D2**	**D1/D2**
0 kA/m (Control)		3	-2.55±0.67	-1.73±0.21	-6.0±10.3 (n = 46)	-13.3±22.1 (n = 53)	NS	NS	NS
61.2 kA/m, 5 min/dose	67.6±16.1 (Low)	4	9.98±3.40	9.30±1.82	-37.4±22.0 (n = 77)	-36.8±17.5 (n = 56)[§]	*****	*****	*****
	98.7±1.9 (High)	3	5.75±4.45[‡]	8.50±3.68[‡]	39.8±32.1 (n = 62)	48.5±66.7 (n = 43)	*****	***	NS
61.2 kA/m, 9 min/dose	79.7 (Low)	1	8.3		-24.6±9.1 (n = 18)		****		
	86.7 (High)	1	14.9		14.6±16.8 (n = 14)		NS		

Abbreviations: *MNPs,* magnetic nanoparticles; MM-OCE, magnetomotive optical coherence elastography; *N*, number of tumor samples; *n*, number of MM-OCE measurements; *B*, before treatment; *D1*, 1st MH dose treatment, *D2*, 2nd MH dose treatment. Statistical significance/insignificance are denoted with asterisks and hyphens, respectively. *****, ****, and *** denote a *p*-value less than 10^{-5}, 10^{-4}, and 10^{-3}; "*NS*" denotes *p*-value > 0.01.

[†]Two samples are excluded from analysis – one without successful delivery of MNPs to the tumor core; the other with MNPs located in a highly heterogeneous tumor microenvironment (**Figure S2**).

[‡]Temperature measurement performed only on 2 out of 3 mice.

[§]Only 3 out of 4 mice were MH-treated for the 2nd time.

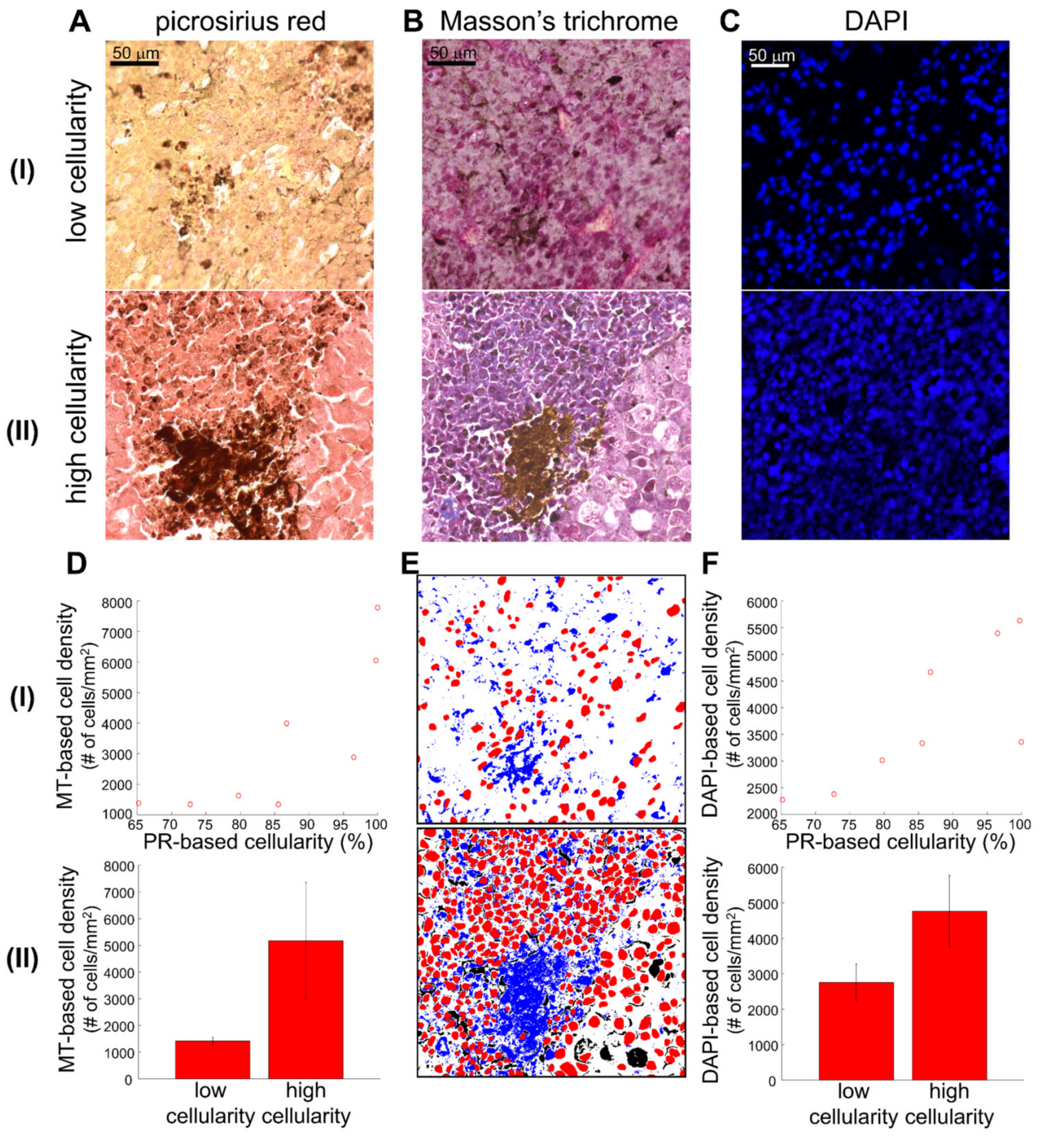

Figure S1. Correlation between picrosirius red (PR) color-based cellularity metrics and cell density (nuclei count per unit area) quantified based on Masson's trichrome (MT) and DAPI. A-C. Representative images of low **(I)** and high **(II)** cellularity tumor sections stained with PR **(A)**, MT **(B)**, and DAPI **(C)**. **D.** PR-based cellularity correlates well with the cell density quantified based on MT histology (Pearson's $r = 0.789$) **(I)**. The cell density of the high cellularity group ($5.2 \pm 1.0 \times 10^3$ counts/mm^2) was higher than that of the low cellularity group ($1.4 \pm 0.1 \times 10^3$ counts/mm^2) (two-sample t-test $p = 0.014$) **(II)**. The nuclei were stained as dark purple in MT histology, **B (I, II)**, whose representative nucleus (*red*) and MNP (*blue*) segmentation was shown in **E (I, II)**, respectively. **F.** PR-based cellularity also correlates well with cell density quantified based on DAPI histology (Pearson's $r = 0.786$) **(I)**. The cell density of the high cellularity group ($4.8 \pm 1.0 \times 10^3$ counts/mm^2) was higher than that of the low cellularity group ($2.8 \pm 0.5 \times 10^3$ counts/mm^2) (two-sample t-test $p = 0.013$) **(II)**. The correlation analyses **(D, F)** were conducted on 8 AMF-treated tumors (11 in total, excluding one that showed absence of MNPs at tumor core, one that had MNPs trapped in a highly heterogeneous environment, **Figure S2**, and one severely necrotic tumor with limited histological sections, **Figure 2B**).

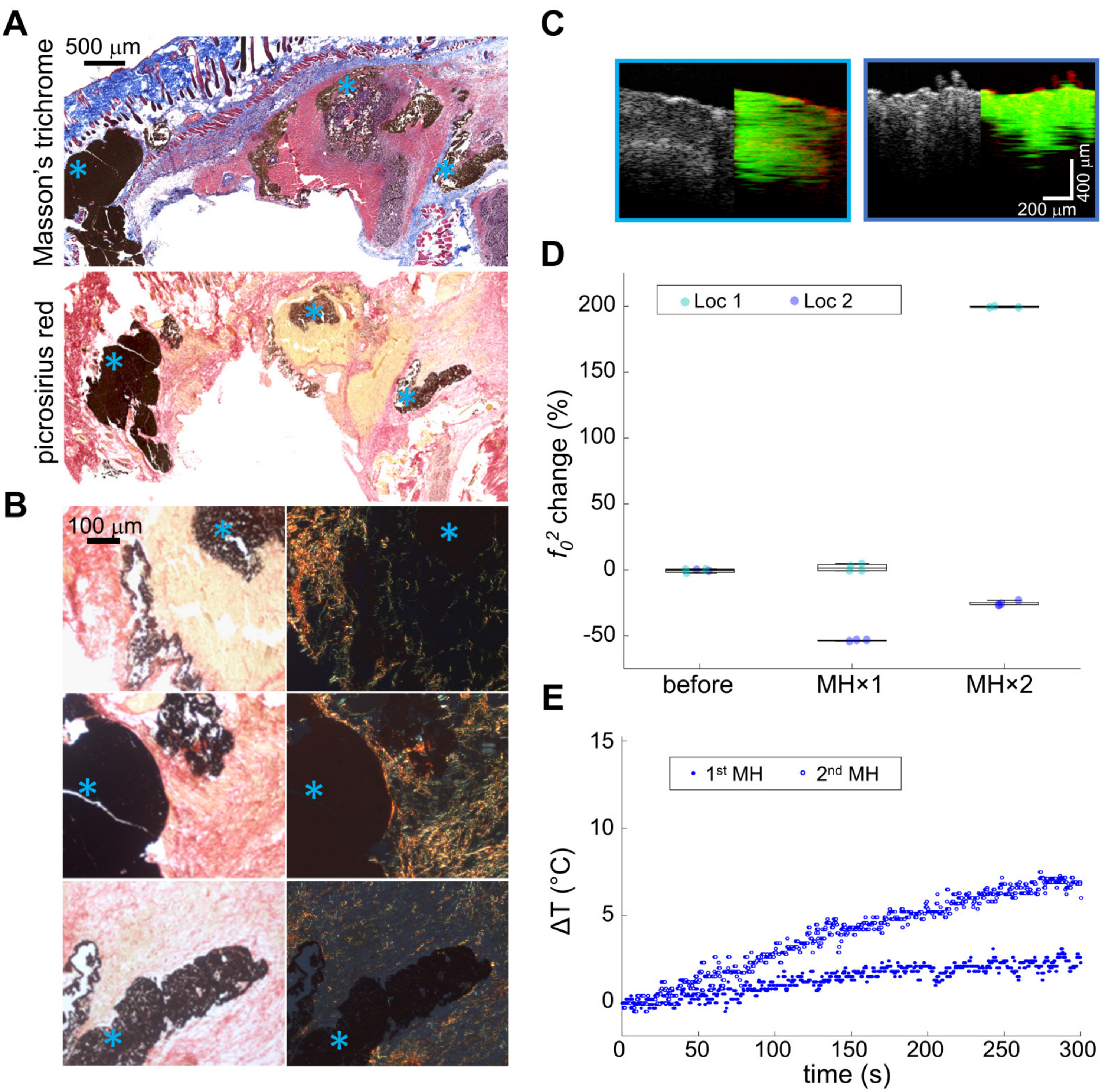

Figure S2. Results from an MNP-injected melanoma tumor with MNPs present both at the tumor center and in the epidermis. A. Images of Masson's trichrome (MT, *upper panel*) and picrosirius red (PR, *bottom panel*)-stained slides visualize the complex environment around the MNPs (*blue asterisks*) with either high or low local cellularity at the tumor site or immediately adjacent to collagen structures. **B.** Enlarged area from the PR-stained slide in **(A)** imaged with both brightfield (*left panel*) and polarized (*right panel*) light. Blue asterisks indicate MNPs. **C.** Optical coherence tomography (OCT, grey-scale) and the corresponding magnetomotive optical coherence tomography (MM-OCT, green and red) images obtained from two representative locations (Loc), where significant MM-OCT signals were observed at both sites, but **D.** the corresponding magnetomotive optical coherence elastography (MM-OCE) results exhibited noticeable differences, potentially suggesting sensitivity of MM-OCE to the local tumor environment near MNPs. **E.** The low rise of temperature after magnetic hyperthermia (MH) treatments.
